# Network Analysis of Emotional Intelligence Dimensions and Related Psychological Resources in Military Personnel

**DOI:** 10.3390/healthcare14132024

**Published:** 2026-07-07

**Authors:** José Gabriel Soriano-Sánchez, Sylvia Sastre Riba

**Affiliations:** 1 Department of Science Didactics, Faculty of Humanities and Education Sciences, University of Jaén, 23071 Jaén, Spain; 2 Department of Educational Sciences, Faculty of Letters and Education, University of La Rioja, Street Luis Ulloa, 2, 26004 Logroño, Spain; silvia.sastre@unirioja.es

**Keywords:** emotional intelligence, network analysis, resilience, mental health, military personnel

## Abstract

**Background:** Psychological adaptation in high-demand contexts such as the military depends on the interaction of multiple emotional and psychological resources. Previous research has mainly examined emotional intelligence, resilience, and self-esteem using latent variable approaches, limiting understanding of how these variables dynamically interact within a broader network of interacting psychological resources. **Objective:** The present study aimed to analyze the network of relationships among emotional intelligence dimensions, resilience, and self-esteem, identifying the most central variables, their degree of clustering, and the strength of their associations. **Methods:** A cross-sectional study was conducted with 739 Spanish military personnel (*M* = 33.29; *SD* = 7.48). A regularized partial correlation network was estimated using the EBICglasso method (γ = 0.5). Centrality indices (strength and expected influence), clustering coefficients, and node predictability were analyzed. Network accuracy and stability were assessed through bootstrap procedures. **Results:** The estimated network showed moderate connectivity, indicating meaningful interrelations among emotional intelligence dimensions, resilience, and self-esteem. General mood and adaptability emerged as the most central nodes within the network. Resilience showed strong positive associations with adaptability and general mood, whereas self-esteem occupied a more peripheral position. Clustering analyses revealed a cohesive organization among adaptive emotional resources. **Conclusions:** Emotional intelligence dimensions and related psychological resources can be conceptualized as a dynamically interacting system associated with emotional adaptation in military personnel. The identification of central components may contribute to the development of targeted interventions aimed at strengthening emotional regulation and psychological adaptation in high-demand environments.

## 1. Introduction

Psychological well-being in high-demand contexts has become a major concern within the field of health, particularly among military personnel who are routinely exposed to operational stress, uncertainty, and emotionally demanding situations [1,2]. In such environments, psychological adaptation is essential for both well-being and operational performance [3,4]. Previous research has consistently identified emotional intelligence, resilience, and self-esteem as important psychological resources associated with emotional regulation, stress adaptation, and psychological adjustment under demanding conditions [5,6,7].

Emotional intelligence is a multidimensional construct that involves the perception, understanding, and regulation of emotions [5]. Its dimensions, including intrapersonal functioning, interpersonal functioning, stress management, adaptability, and general mood, have been linked to adaptive coping and effective emotional regulation under stress [8]. Resilience refers to the capacity to maintain or regain psychological functioning in the face of adversity [6], whereas self-esteem reflects an individual’s overall evaluation of self-worth and personal value [7,9]. Although these constructs are theoretically distinct, they are closely related and may interact dynamically within broader processes of emotional adaptation and stress regulation [10]. Furthermore, higher levels of these psychological resources have been associated with healthier behavioral outcomes, including lower alcohol consumption [11].

A broader theoretical rationale for examining these variables jointly can be found in resource-based models of psychological adaptation. From this perspective, emotional intelligence, resilience, and self-esteem can be conceptualized as complementary psychological resources that contribute to successful adaptation under stressful conditions. Rather than operating independently, these resources are expected to interact and reinforce one another, collectively supporting emotional regulation, coping, and psychological adjustment. This perspective is particularly relevant in military settings, where adaptation to operational demands depends on the coordinated functioning of multiple psychological resources rather than on any single characteristic in isolation. Consequently, a network approach may be especially useful for examining how these resources are interconnected within a broader adaptive system.

Despite the growing body of evidence supporting the role of emotional intelligence, resilience, and self-esteem in psychological adjustment, most studies have examined these variables either independently or through latent variable approaches. Traditional latent models assume that observed indicators reflect underlying common causes and therefore focus primarily on identifying general psychological dimensions [12,13]. Although this perspective has provided valuable insights, it offers limited information regarding how psychological resources interact with one another and contribute collectively to emotional adaptation. In particular, latent variable models do not explicitly account for reciprocal relationships among psychological components, potentially obscuring the mechanisms through which adaptive functioning emerges in real-world contexts [14].

To address these limitations, network analysis has emerged as an alternative theoretical and methodological framework that conceptualizes psychological phenomena as systems of mutually interacting components rather than manifestations of latent entities [12]. From this perspective, emotional adaptation is understood as the result of dynamic interactions among emotional intelligence dimensions and related psychological resources. Network analysis allows researchers to identify structural properties such as centrality, clustering, and patterns of interdependence among variables, thereby providing a more detailed understanding of how adaptive systems are organized [15,16,17,18]. Importantly, centrality does not imply causality but rather reflects the degree to which a node is structurally embedded within the network [14].

This approach is particularly relevant in military contexts. Military personnel are regularly exposed to chronic stress, uncertainty, and demanding operational environments that may increase vulnerability to emotional difficulties and psychological maladjustment [19,20]. Consequently, emotional intelligence, resilience, and self-esteem have been recognized as key protective resources that support adaptation, emotional regulation, and operational effectiveness under sustained stress [21,22]. Previous studies have also suggested that psychological functioning in military populations may be better understood as a complex and interconnected system rather than as a collection of isolated constructs [23]. However, research simultaneously integrating emotional intelligence dimensions, resilience, and self-esteem within a single network model remains scarce.

Building on this framework, the present study adopts a systems-based perspective to examine how emotional intelligence dimensions, resilience, and self-esteem are structurally organized and interconnected within a network of adaptive psychological resources in military personnel. Rather than considering these variables as isolated predictors, the study conceptualizes emotional adaptation as emerging from reciprocal interactions among interconnected psychological resources. This perspective extends previous research linking emotional intelligence to well-being and stress regulation by focusing on the structural organization of adaptive processes within a network framework [13,24].

The aim of the study was to analyze the network relationships among emotional intelligence dimensions, resilience, and self-esteem in military personnel, identifying the most central components, their degree of clustering, and the strength of their associations. Beyond identifying central nodes, this study seeks to provide a more comprehensive understanding of how adaptive psychological resources interact and contribute to emotional adaptation in high-demand contexts. From a network perspective, variables associated with emotional regulation and adaptive functioning are expected to occupy more central positions because they maintain multiple reciprocal connections with other psychological resources within the broader network structure [12,13,14,15,16]. Consistent with emotional intelligence models emphasizing mood regulation and adaptability [5], as well as resilience theory highlighting adaptive coping processes under stress [6,25], emotional adaptation is conceptualized as emerging from dynamic interactions among interconnected psychological resources. Based on this theoretical framework and previous empirical literature on emotional regulation, resilience, and psychological adaptation under stress, three hypotheses (H) were proposed:

**H1.**  *Emotion regulation variables (i.e., general mood, adaptability, and stress management) will show higher centrality within the network.*

**H2.**  *Resilience will show positive associations with emotional intelligence dimensions related to emotional adaptation.*

**H3.**  *The network will show a clustered structure characterized by positive interconnections among adaptive psychological resources.*

Finally, this study contributes to the literature by applying a network approach in an underexplored context and by advancing a systems-based conceptualization of emotional adaptation. Specifically, it provides an integrative framework in which emotional adaptation is understood as emerging from reciprocal interactions among emotional intelligence dimensions and related psychological resources. The findings may help identify structurally relevant components that could inform future intervention strategies aimed at strengthening emotional regulation and psychological adaptation in military personnel operating under high-demand conditions.

## 2. Materials and Methods

### 2.1. Study Design and Participants

This study followed a cross-sectional descriptive design in accordance with the STROBE guidelines [26]. The sample consisted of 739 military personnel from the Spanish Army (officers, non-commissioned officers, and professional soldiers and sailors) stationed at the same military base (Almería), including both operational and support units.

The mean age of participants was 33.29 years (*SD* = 7.48), ranging from 18 to 66 years. The sample comprised 87.70% men (*n* = 648) and 12.30% women (*n* = 91), with mean ages of 33.12 (*SD* = 7.58) and 34.48 (*SD* = 6.64), respectively. Regarding years of military service, 180 participants had up to 5 years of service, 200 between 6 and 10 years, 256 between 11 and 20 years, and 103 between 21 and 40 years. The sample size was determined based on accessibility to military personnel and the operational constraints of the study context. Although no a priori power analysis was conducted, this approach is consistent with current recommendations in psychological network analysis, which emphasize the stability and accuracy of network estimates over traditional power calculations [16,27]. In the present study, bootstrap procedures and centrality stability coefficients indicated acceptable levels of accuracy and stability of the estimated network. Methodological recommendations further indicate that sample adequacy in network models depends not only on the number of participants but also on factors such as the number of nodes, network density, edge strength, and the stability of the estimated parameters [16,27]. Specifically, the present network comprised seven nodes, and bootstrap procedures together with centrality stability coefficients indicated acceptable levels of accuracy and stability of the estimated network, supporting the adequacy of the sample for the analyses performed.

### 2.2. Measures

The instruments used were as follows:

(a) Sociodemographic variables

An ad hoc questionnaire was used to collect sociodemographic information, including gender, age, and years of military service.

(b) Resilience

Was assessed using the *Resilience Scale* (RS) [28], a 25-item instrument designed to evaluate psychosocial adaptation to adversity. Resilience is conceptualized as a dynamic process involving the ability to maintain or regain psychological balance in the face of stress and adversity, including perseverance, coping, and recovery capacities. Items are rated on a 7-point Likert scale ranging from 1 (“strongly disagree”) to 7 (“strongly agree”). Example items include: “When I make plans, I stick to them” and “I tend to take things calmly”. In the present study, internal consistency was *α* = 0.86.

(c) Self-esteem

Self-esteem was measured using the *Rosenberg Self-Esteem Scale* [29] revised version [30], consisting of 10 items rated on a 4-point Likert scale (1 = strongly agree to 4 = strongly disagree). Self-esteem is defined as an individual’s overall evaluation of their own worth, reflecting feelings of self-acceptance, confidence, and personal value. In the present study, internal consistency was *α* = 0.80.

(d) Emotional intelligence

Emotional intelligence was assessed using the *Brief Emotional Intelligence Inventory for Senior Citizens* (EQ-i-M20) [31], a Spanish adaptation of the EQ-i:YV [32]. Although the EQ-i-M20 was originally developed and validated in older adult populations, its use in the present study was considered appropriate because the instrument assesses core emotional intelligence dimensions (intrapersonal functioning, interpersonal functioning, stress management, adaptability, and general mood) that are not specific to older adults. Furthermore, previous research has shown adequate psychometric performance of these dimensions across different adult populations. Given the absence of a brief Spanish instrument assessing these five emotional intelligence dimensions simultaneously, the EQ-i-M20 was selected due to its brevity, multidimensional structure, and satisfactory psychometric properties. The instrument includes 20 items rated on a 4-point Likert scale (1 = very rarely true to 4 = frequently true). It comprises five dimensions: intrapersonal, interpersonal, stress management, adaptability, and general mood. Each dimension is assessed using four items, with higher scores indicating higher levels of emotional intelligence in the corresponding domain. The intrapersonal dimension refers to the ability to understand and express one’s own emotions; the interpersonal dimension reflects social awareness and the capacity to establish and maintain interpersonal relationships; stress management refers to the ability to regulate emotional responses and cope effectively with stress; adaptability reflects flexibility and problem-solving in changing situations; and general mood is associated with optimism and overall emotional adaptation. Example items include: “I find it easy to express how I feel” and “I am confident in myself”. In the present study, overall internal consistency was *α* = 0.78. For each dimension: intrapersonal (*α* = 0.57), interpersonal (*α* = 0.80), stress management (*α* = 0.68), adaptability (*α* = 0.81), and general mood (*α* = 0.83).

### 2.3. Procedure

The study was conducted in accordance with ethical standards and the Declaration of Helsinki [33]. The research protocol was first presented to military authorities for approval. Subsequently, meetings were held with unit leaders to explain the study objectives and procedures. Participants were recruited through coordination with military authorities, who granted access to specific units. Subsequently, unit leaders informed eligible personnel about the study during scheduled briefings, and participation was entirely voluntary. Participants were informed both verbally and in writing about the voluntary nature of participation, confidentiality, and data anonymity.

Written informed consent was obtained prior to participation. The questionnaire, composed of validated psychological scales, was administered individually, with an estimated completion time of 25–30 min. Additionally, appropriate measures were in place to address any unexpected incidents during or after data collection. Inclusion criteria included being active military personnel and providing informed consent to participate in the study. Exclusion criteria included incomplete questionnaire responses or inability to complete the assessment.

### 2.4. Data Analysis

A network analysis was conducted by estimating a regularized partial correlation network using the graphical LASSO algorithm [27,34]. The EBICglasso estimator was implemented with a tuning parameter of γ = 0.5, as commonly recommended in psychological network research to balance model sparsity and sensitivity. This value was selected because it provides a compromise between retaining meaningful associations and reducing the likelihood of spurious connections. Lower values of γ generally produce denser networks, potentially increasing the risk of false-positive edges, whereas higher values yield sparser networks and may exclude weaker but potentially meaningful associations.

However, it should be acknowledged that this choice may influence the resulting network structure, particularly with respect to weaker edges, and alternative specifications may yield partially different configurations [27]. Moreover, higher values of γ may lead to over-regularization, potentially excluding meaningful associations. Therefore, the estimated network should be interpreted with caution, and future research is encouraged to examine the robustness of findings across different regularization parameters through sensitivity analyses.

Given the ordinal nature of the data (Likert-type scales), polychoric correlations were computed instead of Pearson correlations, as they provide more accurate estimates of associations between ordinal variables. All variables were standardized prior to analysis.

Prior to network estimation, the distribution of the items was examined by inspecting skewness and kurtosis values. All items showed acceptable levels of skewness and kurtosis (absolute values < 2), indicating no substantial departures from normality. These results supported the use of polychoric correlations for the analysis of the ordinal Likert-type data.

The network was visualized using the Fruchterman–Reingold algorithm. Centrality measures included strength and expected influence. Expected influence was specifically selected because it accounts for both positive and negative edge weights, making it particularly suitable for psychological networks where inhibitory and facilitative relationships coexist. In contrast, metrics such as betweenness and closeness were not interpreted due to their limited stability in regularized psychological networks [16].

To assess network accuracy, nonparametric bootstrap analyses (*n* = 1000) were conducted to estimate confidence intervals for edge weights. The stability of centrality indices was evaluated using the correlation stability coefficient (CS-coefficient), with values above 0.25 considered acceptable.

The analyses were conducted using the statistical software JASP, version 0.96.0 for Windows.

### 2.5. Ethical Considerations

The study was approved by the Ethics Committee of the Central Defense Hospital (Approval code: 51117, 26 October 2017).

## 3. Results

### 3.1. Overview of the Network

The estimated network included 7 nodes and 15 non-zero edges out of 21 possible connections (sparsity = 0.29; Table 1), indicating a moderately connected and partially sparse structure. Bootstrap analyses showed relatively narrow confidence intervals for the main edges, suggesting adequate estimation precision. Centrality stability was acceptable (CS-coefficient = 0.67).

### 3.2. Centrality Measures

Centrality indices revealed differences in node positions within the network (Table 2). General mood and adaptability showed the highest levels of connectivity across centrality metrics, suggesting that these variables occupy structurally prominent positions within the network. Resilience also demonstrated relatively high centrality values, indicating strong integration with other adaptive psychological resources.

In contrast, self-esteem showed comparatively lower centrality values, suggesting a more peripheral position within the network structure. The remaining variables showed intermediate levels of connectivity, indicating a relatively distributed network organization. Importantly, the high centrality of general mood suggests that it may represent a highly connected component linking multiple emotional intelligence dimensions and related psychological resources associated with emotional adaptation.

### 3.3. Clustering Measures

Clustering coefficients varied across nodes (Table 3), indicating heterogeneity in local network organization. Higher clustering values were observed for general mood, resilience, and adaptability, suggesting that these variables form tightly interconnected substructures within the network. Self-esteem and intrapersonal functioning also showed relatively elevated clustering coefficients, indicating meaningful local integration with other adaptive psychological resources. In contrast, interpersonal functioning and stress management displayed comparatively lower clustering values, suggesting weaker local cohesion within the network structure. Overall, the observed pattern is consistent with a partially cohesive organization of emotional intelligence dimensions and related psychological resources associated with emotional adaptation. More specifically, the clustering pattern suggests the existence of a closely interconnected subgroup composed of resilience, adaptability, and general mood. These variables showed consistently elevated clustering coefficients and may represent a local adaptive system associated with emotional regulation, flexible coping, and positive affective functioning. The presence of this subgroup is theoretically consistent with models of emotional adaptation emphasizing the reciprocal interaction of coping resources and emotional regulation processes under stressful conditions. Clustering metrics are presented in detail due to their relevance for interpreting local network structure and interconnectedness among variables.

### 3.4. Network Structure

**Figure 1:**
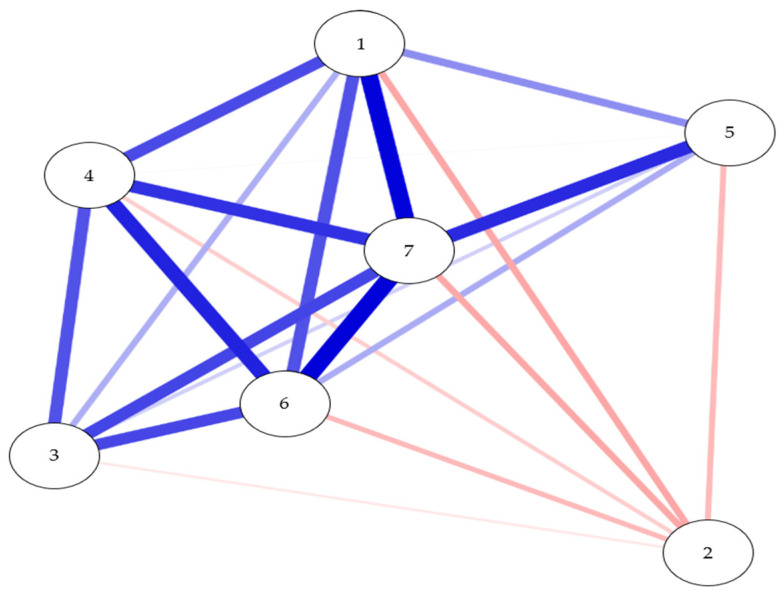
Network structure of emotional variables. Note: 1 = Resilience; 2 = Self-esteem; 3 = Intrapersonal; 4 = Interpersonal; 5 = Stress Management; 6 = Adaptability; 7 = General Mood.

The network structure (Figure 1) showed that general mood and adaptability occupied central positions within the network, whereas self-esteem appeared relatively more peripheral. The spatial configuration of the network supports the presence of a cohesive cluster of adaptive psychological resources, particularly among emotional intelligence dimensions related to emotional regulation and adjustment. Strong positive associations were observed among resilience, adaptability, intrapersonal functioning, interpersonal functioning, and general mood, indicating a highly interconnected adaptive structure.

In addition to their central positions within the network, general mood and adaptability showed the strongest positive associations with other adaptive psychological resources, particularly resilience, intrapersonal functioning, and interpersonal functioning. These connections suggest that positive affective functioning and adaptive coping processes may serve as important organizational elements linking multiple components of emotional adaptation. In contrast, self-esteem showed comparatively weaker and partially negative associations with several variables, suggesting lower integration within the overall network configuration. These findings are consistent with the clustering and centrality results, reinforcing the interpretation of emotional adaptation as a dynamically interconnected system.

The most prominent edge weights within the network are presented in Table 4. 

The strongest positive associations were observed between stress management and general mood (w = 0.257), resilience and general mood (w = 0.236), adaptability and general mood (w = 0.235), and adaptability and interpersonal functioning (w = 0.189). Additional positive associations were found between resilience and interpersonal functioning (w = 0.144), adaptability and intrapersonal functioning (w = 0.144), and intrapersonal functioning and general mood (w = 0.143).

In contrast, self-esteem showed comparatively weaker and predominantly negative associations with several variables, suggesting lower integration within the overall network structure. The strongest negative edge weights were observed between self-esteem and resilience (w = −0.069), self-esteem and stress management (w = −0.047), and self-esteem and general mood (w = −0.045).

## 4. Discussion

The present study provides evidence regarding the structural organization of emotional intelligence dimensions and related psychological resources in military personnel operating in high-demand contexts. Consistent with the network approach in psychology, the findings support the idea that emotional adaptation emerges from dynamic interactions among interconnected psychological resources rather than from isolated or independent components. This perspective is particularly relevant in military environments, where emotional regulation is closely associated with operational performance, decision-making under pressure, and psychological adjustment under sustained stress.

From a theoretical standpoint, these findings support a systems-based perspective in which emotional intelligence dimensions interact dynamically with related psychological resources such as resilience and self-esteem. Rather than conceptualizing these variables as indicators of a single latent construct, the present results suggest that emotional adaptation may emerge from reciprocal interactions among partially distinct but interconnected psychological components. In this sense, the study extends previous research by applying network analysis to examine how emotional intelligence dimensions are structurally embedded within the broader network of interacting psychological resources [12,14].

Regarding H1, the results indicate that variables associated with emotional regulation occupied relatively central positions within the network. In particular, general mood and adaptability emerged as the most central nodes, suggesting that positive affective functioning and flexible emotional regulation may represent structurally prominent components within the network structure. These findings are consistent with emotional intelligence models emphasizing the role of mood regulation and adaptability in stress management, cognitive appraisal, and psychological adjustment [5]. From a network perspective, however, centrality should not be interpreted as evidence of causal influence. Rather, highly central nodes reflect components that are strongly interconnected with other variables within the system [16]. Consequently, general mood and adaptability may represent important organizational elements within the network without necessarily constituting direct causal mechanisms.

Stress management showed moderate connectivity within the network. This finding is theoretically meaningful in military contexts characterized by chronic operational demands and sustained stress exposure. The stress management dimension may reflect both perceived regulatory capacity and the subjective experience of coping difficulties, which could explain its broad pattern of associations with other adaptive resources. Importantly, its central position within the network suggests that stress regulation processes are closely intertwined with broader mechanisms of emotional adaptation in military personnel [35,36]. Nevertheless, this interpretation should remain cautious given the cross-sectional nature of the data and the conceptual complexity of stress regulation measures.

Regarding H2, resilience showed strong positive associations with emotional intelligence dimensions related to emotional adaptation, particularly adaptability and general mood. This finding is consistent with resilience theory, which conceptualizes resilience as a dynamic process involving adaptive coping, emotional regulation, and psychological recovery under adverse conditions [6]. Within the network structure, resilience appeared highly integrated with adaptive emotional resources, supporting the idea that resilience does not operate independently but rather in coordination with broader emotional regulation processes. This pattern reinforces previous evidence suggesting that resilient functioning in military populations depends on the interaction of multiple psychological resources rather than on isolated protective traits [21,22,37].

Regarding H3, the findings revealed a clustered structure characterized by positive interconnections among adaptive psychological resources. In particular, resilience, adaptability, intrapersonal functioning, interpersonal functioning, and general mood formed a cohesive and highly interconnected subgroup within the network. This pattern suggests that emotional adaptation in military contexts may depend on coordinated interactions among emotional regulation, flexible coping, positive affect, and interpersonal functioning. At the same time, self-esteem occupied a relatively more peripheral position within the network, indicating lower structural integration compared with other adaptive variables. Although self-esteem remains an important psychological resource, its role within the network appeared less central than dimensions directly associated with emotional regulation and adaptation.

An alternative explanation for the relatively peripheral position of self-esteem may be related to measurement and sample characteristics. The Rosenberg Self-Esteem Scale assesses a broad and relatively stable evaluation of self-worth [38], whereas emotional intelligence dimensions and resilience are more directly linked to context-dependent emotional regulation and adaptive functioning. Consequently, self-esteem may show weaker partial associations within a network specifically focused on emotional adaptation processes. Additionally, the characteristics of the present military sample may have influenced this pattern. Military environments often emphasize discipline, collective identity, and operational performance, potentially reducing the relative importance of individual self-evaluative processes within the broader system of adaptive psychological resources. Future research should examine whether similar network configurations are observed in more diverse occupational and civilian populations.

From a broader theoretical perspective, these findings reinforce the value of network analysis for examining emotional adaptation in high-demand contexts. By identifying structurally prominent variables and patterns of local organization, this approach provides insights into how adaptive psychological resources interact within a broader emotional system [12,13]. Nevertheless, several methodological challenges should be acknowledged. In particular, the interpretation of centrality metrics remains subject to debate, as these indices may vary depending on sample characteristics, measurement properties, and estimation procedures [16]. Furthermore, although the selected γ value is commonly recommended in psychological network research, the absence of sensitivity analyses examining alternative regularization parameters limits the ability to evaluate the robustness of the observed structure across different model specifications.

From an applied perspective, the identification of highly connected nodes such as general mood, adaptability, and stress management may provide a preliminary framework for future intervention development in military populations. However, given the cross-sectional nature of the network, these findings should be considered hypothesis-generating rather than evidence of causal influence. Longitudinal and experimental studies are needed to determine whether changes in highly connected variables produce broader changes across the emotional system. Second, the exclusive use of self-report measures may introduce response biases, particularly in military contexts where social desirability and performance-related norms may influence emotional reporting. Military personnel may be less willing to disclose emotional difficulties, stress-related experiences, or perceived vulnerabilities due to concerns about professional image, unit cohesion, or operational readiness. Consequently, some psychological resources and emotional characteristics may have been overestimated or underestimated, potentially influencing the observed network structure.

Future studies would benefit from incorporating multimethod assessment strategies, including behavioral indicators, peer evaluations, physiological measures, or clinician-administered assessments, to provide a more comprehensive understanding of emotional adaptation in military populations. Third, although all scales demonstrated acceptable internal consistency coefficients for psychological research, measurement limitations inherent to self-report instruments should still be acknowledged. Although the reliability estimates obtained in the present study were within acceptable ranges, differences in internal consistency across measures may have influenced the strength of some associations within the network. Measurement error can attenuate relationships among variables, potentially affecting the magnitude of edge weights and the relative position of nodes within the network structure. Therefore, the findings should be interpreted in light of the psychometric properties of the instruments employed. Fourth, network estimation depends on methodological decisions, including regularization parameters and model specification, and alternative estimation approaches may produce partially different network structures. Furthermore, although the sample size exceeded those commonly reported in many psychological network studies, there is currently no universal consensus regarding optimal sample size requirements for network estimation. The accuracy and stability of network parameters depend on multiple factors, including network density, effect sizes, measurement quality, and the number of nodes included in the model. Consequently, sample adequacy in network analysis should be evaluated not only in terms of participant numbers but also through indicators of network accuracy and stability.

In the present study, bootstrap analyses and centrality stability coefficients suggested acceptable levels of precision and stability; however, replication in independent samples remains necessary to confirm the robustness of the observed network structure. Finally, several sample-related limitations should be acknowledged. Participants were recruited from a single military base in Spain, which may limit the generalizability of the findings to other military units, operational contexts, or national armed forces. In addition, the sample was predominantly male, reflecting the demographic composition of the military population studied. Although this distribution is representative of many military settings, it restricts the ability to generalize the findings across gender groups and may have influenced the observed network structure. Future studies should include more geographically diverse military samples and greater gender representation to examine the stability and generalizability of the network configuration.

Despite these limitations, the present study makes several important contributions. First, it provides an integrative system-level perspective on emotional adaptation by simultaneously examining emotional intelligence dimensions, resilience, and self-esteem within a unified network model. Second, it extends previous research by applying network analysis to a high-demand population, offering novel insights into the structural organization of adaptive psychological resources in military contexts. Third, the identification of central and peripheral components contributes to a more nuanced understanding of how emotional regulation processes are organized within broader adaptive systems. Nevertheless, the interpretation of these findings should take into account the characteristics of the study sample, which was drawn from a single military base and predominantly composed of men. Consequently, replication in more diverse military populations is needed before broader conclusions can be established. Finally, the findings generate theoretically informed hypotheses that may guide future longitudinal and intervention-based research.

Future research should employ longitudinal and dynamic designs to examine how emotional networks evolve over time and across operational contexts in military populations. The inclusion of multimethod assessments and alternative network estimation procedures would also strengthen the robustness and validity of future findings. Additionally, future studies should explore whether network structures differ according to gender, military rank, service branch, or exposure to operational stressors.

## 5. Conclusions

The present study provides evidence supporting the conceptualization of emotional intelligence dimensions, resilience, and self-esteem as interconnected psychological resources associated with emotional adaptation in military personnel. Rather than operating as isolated variables, these psychological resources appear to maintain reciprocal relationships that contribute to adaptive functioning under high-demand conditions.

Within the network structure, general mood and adaptability emerged as the most central components, suggesting that positive affective functioning and flexible emotional regulation may play an important organizational role within the adaptive system. Resilience also showed strong integration with emotional intelligence dimensions, reinforcing the idea that psychological adaptation in military contexts depends on coordinated interactions among multiple adaptive resources.

These findings highlight the usefulness of network analysis for understanding emotional adaptation from a systems-based perspective, overcoming some limitations of traditional latent variable approaches. At the same time, the findings should be interpreted cautiously, as cross-sectional network structures do not provide evidence of causality or direct intervention effects.

Overall, the study contributes to the growing literature applying network analysis to psychological functioning in high-demand contexts and provides a theoretically grounded framework for future longitudinal and intervention-based research in military populations.

## Figures and Tables

**Table 1:** Network summary.

Number of Nodes	Number of Non-Zero Edges	Sparsity
7	15/21	0.29

**Table 2:** Centrality measures.

Network		
Variable	Strength	Expected Influence
Resilience	0.88	0.84
Self-esteem	0.61	0.58
Intrapersonal	0.76	0.72
Interpersonal	0.69	0.66
Stress Management	0.67	0.63
Adaptability	1.02	0.98
General Mood	1.34	1.29

**Table 3:** Clustering measures.

	Network			
Variable	Barrat ^a^	Onnela	WS ^a^	Zhang
Resilience	0.62	0.88	0.71	0.74
Self-esteem	0.58	0.65	0.69	0.81
Intrapersonal	0.55	0.72	0.63	0.79
Interpersonal	0.49	0.61	0.57	0.68
Stress Management	0.53	0.70	0.60	0.73
Adaptability	0.57	0.76	0.66	0.77
General Mood	0.60	0.91	0.74	0.83

Note: ^a^ = Barrat and WS refer to weighted clustering coefficients calculated according to their respective formulations.

**Table 4:** Most prominent edge weights in the estimated network.

Edge	Weight (w)
Stress Management—General Mood	0.25
Resilience—General Mood	0.23
Adaptability—General Mood	0.23
Adaptability—Interpersonal Functioning	0.18
Resilience—Interpersonal Functioning	0.14
Adaptability—Intrapersonal Functioning	0.14
Intrapersonal Functioning—General Mood	0.14
Self-Esteem—Resilience	−0.06
Self-Esteem—Stress Management	−0.04
Self-Esteem—General Mood	−0.04

Note: Only the largest positive and negative edge weights are presented to facilitate the interpretation of the network structure. Positive values indicate positive associations between nodes, whereas negative values indicate inverse associations. Higher absolute values represent stronger connections within the regularized partial correlation network.

## Data Availability

The data presented in this study are available upon reasonable request from the corresponding author. They are not publicly available due to ethical and privacy restrictions.
